# Illness perceptions among individuals with endometriosis and their longitudinal associations with psychological distress and pain

**DOI:** 10.1177/13591053251320595

**Published:** 2025-02-24

**Authors:** Chloe Moore, Nicola Cogan, Lynn Williams

**Affiliations:** University of Strathclyde, Glasgow, UK

**Keywords:** illness perceptions, endometriosis, anxiety, depression, pain

## Abstract

This study examined the illness perceptions held by individuals living with endometriosis, and their associations with psychological distress and chronic pain, over time. At baseline, 408 participants provided demographic and clinical information and completed measures of illness perceptions, anxiety and depression, and pain. One-year later, 283 of these participants completed the same measures again. Results showed that participants held largely negative perceptions of their endometriosis, perceiving adverse consequences, enduring timeline, and negative emotional representations of their condition. Additionally, participants felt a lack of personal control over the condition. Multiple regression analyses (controlling for demographics, clinical factors, and baseline levels of the outcome variables) showed that illness perceptions do not predict anxiety and depression at 12-month follow-up. However, the perception of illness timeline did significantly predict pain intensity at follow-up.

## Introduction

Endometriosis is a chronic, incurable condition in which tissue similar to the lining of the womb, the endometrium, binds to organs and tissue outside the uterus (Zondervan et al., 2020). One in ten women and individuals assigned female at birth are diagnosed with endometriosis (World Health Organization, 2023). Recurrent pelvic pain is a significant symptom of endometriosis. Pelvic pain may be constant or cyclical, occurring most frequently around the time of menstruation and ovulation (Drabble et al., 2021). Alongside menorrhagia (painful, often heavy periods), pain-related symptoms commonly associated with endometriosis include dyspareunia (pain during sexual activity), bladder pain (often coupled with recurrent urinary tract infections), and dyschezia (painful defecation) (Montanari et al., 2019). Affected individuals have also described the presence of pain in their legs, back and joints (Drabble et al., 2021; Young et al., 2015). Alongside pain, symptoms typical of endometriosis include abdominal bloating, persistent fatigue, and low mood (World Health Organization, 2023).

Depression and anxiety are the most common psychological conditions associated with endometriosis (Pope et al., 2015). Estimates of the prevalence of depression and anxiety for those experiencing endometriosis are as high as 86.5% and 87.5% respectively (Sepulcri and Amaral, 2009). A meta-analysis of 44 articles from 13 countries identified significant differences in depression and anxiety risk between individuals with endometriosis symptoms and symptom-free controls (Wang et al., 2021). Individuals with endometriosis were consistently more likely to present with anxiety and depression than control groups, regardless of the country of origin of the research or the methods used to assess depression and anxiety symptoms.

When individuals are confronted with an illness, they create their own models and representations of the illness to help them make sense of, and respond to, the problems they are faced with. Leventhal’s Self-Regulation Model (1997) offers a comprehensive framework for understanding how individuals perceive and respond to health threats. The model suggests that individuals engage in a series of cognitive and emotional processes to interpret health threats and initiate adaptive coping strategies. Central to the model is the concept of illness representations (or illness perceptions) wherein individuals construct cognitive schemas about their health conditions based on various factors including perceptions of the consequences, controllability, causation, and the timeline of the health threat or condition. These representations, in turn, influence individuals’ emotional responses and coping behaviors aimed at managing the health threat (Leventhal et al., 1997).

Illness perceptions are associated with health and wellbeing outcomes across a range of health conditions, including breast cancer (Fanakidou et al., 2018), irritable bowel syndrome (Knowles et al., 2017) and fibromyalgia (Homma et al., 2018). Generally, more positive illness perceptions relate to more positive outcomes, and negative illness perceptions result in more negative health and wellbeing experiences (Dempster et al., 2015). Illness perceptions have been linked to health-related behaviors including medication adherence, (Shiyanbola et al., 2018), health outcomes such as mortality, health-related quality-of-life (Knowles et al., 2020), mental distress (Rochelle and Fidler, 2013) and coping strategies (Woodhouse et al., 2018). Based on this evidence, interventions aimed at addressing illness perceptions have been developed with people experiencing a range of conditions, such as myocardial infarction (Sararoudi et al., 2016), type 2 diabetes (Alyami et al., 2021), and chronic back pain (Siemonsma et al., 2013).

The impact of endometriosis on psychological distress and chronic pain is now well-established, but less is known about the illness perceptions of those living with endometriosis or about the relationship between illness perceptions and the key outcomes of psychological distress or chronic pain. Recently, Moore et al. (2023) undertook a qualitative investigation into the role of illness perceptions in endometriosis. They found that the illness perceptions held by individuals experiencing endometriosis were complex and dynamic. Endometriosis-specific symptoms such as pain were the main driver of reduced quality of life, and these symptoms and their associated impact cultivated and molded endometriosis-related illness perceptions. In addition, another recent study by Barberis et al. (2023) also pointed to the potential importance of illness perceptions in endometriosis by identifying cross-sectional associations between illness perceptions, quality of life, and psychological distress in women with a clinical diagnosis of endometriosis.

The present study aims to extend previous research by longitudinally examining the associations between illness perceptions and key outcomes in endometriosis (i.e. psychological distress and chronic pain). There are three key research aims: (a) to examine what illness perceptions are held by individuals with endometriosis; (b) to examine the relationship between illness perceptions and psychological distress, over time, and (c) to examine the relationship between illness perceptions and pain intensity, and pain-related disability, over time. It was hypothesized that the perceptions of concern and emotional response would longitudinally predict anxiety and depression, and perceptions of timeline, consequences and control would predict pain.

## Materials and methods

### Participants and procedure

Participants (*n* = 408) accessed the survey predominantly through endometriosis support groups (37.2%) and social media platforms (36.9%). Individuals aged over 18, residing in the UK or Ireland, and who self-reported a medically confirmed diagnosis of endometriosis were eligible to participate. Two hundred and eighty-three participants completed the follow-up survey 12 months’ later, indicating an attrition rate of 30.8%. Analyses demonstrated no significant differences between those who completed the follow-up survey and those who did not in demographic background or health status. As shown in Table 1, participants were aged between 19 and 56 with a mean age of 33.9 years (SD: 8.0). The majority of participants were from White backgrounds (93.6%) and were in full or part time employment (72.1%). Participants had experienced endometriosis for approximately 15.5 years (SD: 8.44) and had been diagnosed for around 5.07 years (SD = 5.6). Most participants disclosed at least one co-morbid condition (57.6%) and had undergone surgery for endometriosis (86.6%).

**Table 1.** Sample information at baseline and follow-up.

Characteristic	Total at baseline	Total at follow-up
Age, *M* (SD), y	33.92 (7.99)	—
Household income (Mode)	£20,000– £29,000	—
University education, %	61.5	—
Employed full-time, %	59.3	—
Years since symptom onset, *M* (SD), y	15.5 (8.44)	—
Years since diagnosis, *M* (SD), y	5.07 (5.61)	—
Undergone surgery, %	84.1	—
Comorbid condition, %	58.8	—
Depression, *M* (SD)	2.83 (1.84)	2.57 (1.87)
Anxiety, *M* (SD)	3.1 (1.88)	2.92 (1.93)
Pain intensity, *M* (SD)	67.21 (18.16)	60.54 (23.19)
Pain-associated disability, *M* (SD)	3.96 (1.92)	3.17 (2.13)
Consequences	7.79 (1.92)	6.98 (2.34)
Timeline	8.96 (1.95)	9.0 (2.01)
Identity	7.56 (2.09)	6.88 (2.48)
Concern	7.9 (2.17)	7.14 (2.5)
Emotional representation	8.11 (2.12)	7.49 (2.4)
Personal control	2.46 (2.32)	2.78 (2.45)
Treatment control	4.98 (2.43)	5.15 (2.32)
Coherence	7.37 (2.46)	7.42 (2.27)

Ethical approval was obtained from the University’s Ethics Committee. Both baseline and follow-up surveys were hosted online through Qualtrics. For both phases of the survey, participants were first presented with an information sheet detailing the aims and scope of the study. They were then asked to indicate their consent to participate. During the baseline survey, participants were asked to confirm that they had received a diagnosis of endometriosis—if they indicated that they were not medically diagnosed, they were directed to the end of the survey. Subsequently, participants completed the baseline survey before receiving a debrief. Twelve months after participating in the baseline survey, the follow-up survey was distributed via email to participants using the contact details provided in the baseline survey. Participants were required to read another information sheet and reiterate their consent to participate. Following completion of the survey, participants were debriefed and given links to various endometriosis websites, along with mental health resources in case the survey had any emotional impact.

### Measures

#### Demographic and clinical information

At baseline, we collected demographic information on age, educational attainment, employment status, and household income, together with clinical information on whether participants experienced co-morbid conditions, their history of endometriosis surgery, and the duration of their endometriosis symptoms and diagnosis.

The following self-report measures were completed at baseline and at 1 year follow-up:

#### Illness perceptions

The Brief Illness Perceptions Questionnaire (B-IPQ; Broadbent et al., 2006) was used to measure illness perceptions. It contains nine items which measure the nine dimensions of illness perceptions: identity, timeline, personal control, treatment control, consequences, cause, emotional response, concern, and illness coherence. Capitalising on the flexibility afforded by the B-IPQ, within the present research the term “illness” was replaced with “endometriosis” on all items in the questionnaire. All questions, aside from the “cause” item, were scored on a 11-point Likert scale (e.g. 0: “absolutely no control” —10: “extreme amount of control”). For the “cause” item, participants were asked to list, in rank order, up to three factors they believed to have caused their endometriosis.

#### Pain

The 7-item Chronic Pain Grade (CPG; Von Korff et al., 1992) was used to measure endometriosis-related pain intensity and disability. The measure includes 3 subscales: (i) characteristic pain intensity; (ii) disability score; (iii) disability points. Disability points are derived by combining the number of ‘disability days’ indicated by participants with the disability score derived from the scale. Each item on the CPG is scored on a 11-point Likert scale (e.g. 0 = no pain, 10 = pain as bad as it could be) with the exception of question 4 which asks participants, “about how many days in the past 6 months have you been kept from your usual activities because of your pain?.” This question gauges “disability days,” and asks participants to indicate the approximate number of days in which they’ve been unable to function as a result of their condition. Participants’ disability points were used to represent self-reported disability, whilst characteristic pain intensity was used to represent self-reported pain.

#### Psychological distress

The Patient Health Questionnaire-4 (PHQ-4; Kroenke et al., 2009) was used to assess symptoms of anxiety and depression. The questionnaire combines the Patient Health Questionnaire-2 (PHQ-2; Kroenke et al., 2003), a 2-item assessment of depression, and the General Anxiety Disorder-2 scale (GAD-2; Kroenke et al., 2007), a 2-item measure of anxiety. Although not a diagnostic tool, the PHQ-4 is a reliable indicator of depression and/or anxiety susceptibility in the general population (Löwe et al., 2010). Each of the four items are scored on a 4-point Likert scale (0: “Not at all” —3: “Nearly every day”). Anxiety is calculated by combining the scores of questions 1 and 2, whilst depression is determined by combining the scores of questions 3 and 4. A score of 3 or greater for the first two items indicates anxious mood, whilst a score of 3 or above for questions 3 and 4 suggests depressive symptoms. Both the anxiety (α = .87) and depression (α = .86) subscales demonstrated good reliability.

### Statistical analysis

First, descriptive statistics pertaining to the sample characteristics and illness perceptions were established. Correlations, means, and SDs were then computed for all variables to examine the cross-sectional associations between illness perceptions and the outcome variables (i.e. depression, anxiety, pain intensity, pain-related disability) measured at baseline. Next, hierarchical multiple regression analysis was conducted to determine whether illness perceptions predicted changes in anxiety, depression, pain intensity, and pain-related disability at follow-up, after controlling for initial levels of the outcome variables. Sociodemographic factors were entered at Step 1, followed by clinical characteristics at Step 2. Baseline levels of the outcome variables were entered at step 3, before illness perceptions were entered at Step 4.

## Results

### Sample characteristics

Baseline clinical and demographic information is presented in Table 1, alongside baseline and follow-up values from the psychological measures.

Mean values and standard deviations were calculated for each illness perception dimension (see Table 1). Higher scores indicate more negative illness perceptions, with the exception of the personal control, treatment control, and coherence dimensions, in which lower scores indicate more negative illness perceptions. As illustrated by Table 1, participants held largely negative perceptions of their endometriosis. Participants envisioned adverse consequences associated with endometriosis, identified with many symptoms of the condition, perceived that endometriosis would endure throughout their lifespan, and held negative emotional representations of their condition. Additionally, participants felt a lack of personal control over the condition, although they held neither positive nor negative perceptions over the control of their treatment, perhaps reflecting the heterogeneity of treatment effectiveness for endometriosis. Participants generally reported a strong understanding of their condition.

As part of the B-IPQ, participants were asked whether they perceived a cause associated with their condition. Two hundred and one participants (49.3%) suggested potential causes for their endometriosis. Of these respondents, 137 (68.2%) believed that their condition was primarily caused by genetic and hereditary factors including genes and a family history of endometriosis. Other potential causes suggested by participants included trauma (5.5%), hormones (4.5%), birth control (4.5%), early onset of menstruation (3%), surgery (2.5%), giving birth (2.5%), diet/lifestyle (2%) and immune response (2%). However, the “cause” dimension of the B-IPQ was ultimately dropped from further analysis due to: (i) limited heterogeneity in participant responses; and (ii) the lack of participants who responded to this item.

### Cross-sectional correlation analysis

We examined the cross-sectional correlations between the illness perceptions and outcome variables assessed at baseline. As shown in Table 2, 7 out of 8 of the illness perceptions dimensions were significantly associated with anxiety and depression, with coherence the only dimension that was non-significant. For pain intensity, again coherence was the only dimension that was not significant, while for pain-related disability, treatment control was the only non-significant dimension.

**Table 2.** Cross-sectional correlation analysis at baseline.

Variable	Consequences	Timeline	Personal control	Treatment control	Identity	Concern	Coherence	Emotional response	Anxiety	Depression	Pain Intensity	Pain Disability
Consequences	—	.299**	−.306**	−.206**	.682**	.624**	.081	.597**	.336**	.389**	.631**	.613**
Timeline		—	−.096	−.263**	.224**	.225**	.009	.251**	.154**	.174**	.319**	.183**
Pers control			—	.295**	−.277**	−.349**	.136**	−.286**	−.256**	−.275**	−.256**	−.167**
Treat control				—	−.155**	−.150**	.043	−.194**	−.139**	−.134**	−.182**	−.084
Identity					—	.518**	.087	.451**	.271**	.417**	.683**	.600**
Concern						—	.002	.590**	.304**	.352**	.448**	.403**
Coherence							—	.062	−.039	−.020	.052	.126*
Emotion resp								—	.375**	.415**	.456**	.424**
Anxiety									—	.623**	.325**	.296**
Depression										—	.418**	.398**
Pain intensity											—	.638**
Pain disability												—

* *p* < .05. **p < .01.

### Predictors of psychological distress

Hierarchical multiple regression analyses were then carried out for each of the four outcome variables (i.e. anxiety, depression, pain intensity, pain-related disability). As shown in Table 3, with anxiety as the outcome variable, in the first step demographics accounted for a significant amount of T2 anxiety, *ΔR*^2^ = 0.051, *p* < .01. The inclusion of clinical factors explained only an additional 2.1% of the variance, *ΔR*^2^ = 0.021, ns. Anxiety scores at baseline explained a further 27.5% of the variance in anxiety at follow-up, *ΔR*^2^ = 0.275, *p* < .001. Illness perceptions were entered in the final step and together they explained an additional 1.9% of the variance in anxiety, *ΔR*^2^ = 0.019, ns. In the final model, baseline anxiety (β = .516, *p* < .001) was the only significant predictor.

**Table 3.** Hierarchical regression analyses predicting time 2 anxiety.

Step		β at step	Δ*R*^2^ for step	Total *R*^2^	95% CI
Step 1	Age	−.067			−.044–.012
	Income	−.104			−.132–.011
	Education	−.110			−.929–.045
	Employment	.099	.051**	.051**	−.056–.524
Step 2	Age	−.154			−.074–.001
	Income	−.102			−.131–.013
	Education	−.116			−.950–.024
	Employment	.095			−.069–.518
	Symptom duration	.212*			.008–.088
	Time since diagnosis	−.106			−.085–.015
	History of surgery	−.008			−.722–.629
	Comorbidity	.018	.021	.071*	−.410–.546
Step 3	Age	−.028			−.060–.003
	Income	−.005			−.066–.057
	Education	.053			−.367–.474
	Employment	.123			−.124–.370
	Symptom duration	.015			−.019–.049
	Time since diagnosis	−.004			−.046–.039
	History of surgery	.046			−.522–.614
	Comorbidity	.082			−.320–.484
	Baseline anxiety	.571**	.275***	.347***	.463–.678
Step 4	Age	−.024			−.057–.008
	Income	.000			−.062–.063
	Education	.125			−.318–.567
	Employment	.120			−.130–.369
	Symptom duration	.010			−.026–.046
	Time since diagnosis	−.002			−.045–.041
	History of surgery	.042			−.537–.621
	Comorbidity	.086			−.323–.495
	Baseline anxiety	.516***			.398–.634
	Consequences	.090			−.072–.252
	Timeline	.030			−.079 - .139
	Personal control	−.028			−.119–.064
	Treatment control	.000			−.090–.090
	Identity	−.025			−.152–.102
	Concern	−.043			−.171–.085
	Coherence	−.042			−.124–.040
	Emotional response	.085	.019	.365***	−.042–.212

* *p* < 0.05. **p < 0.01. ***p < 0.001.

For depression as the outcome variable, demographic factors explained a significant amount of the variance, *ΔR*^2^ = 0.064, *p* < .01, and the inclusion of clinical factors explained a further 1.9% of the variance, *ΔR*^2^ = 0.019, ns. Baseline depression scores explained a further 34.4% of the variance in depression at follow-up, *ΔR*^2^ = 0.344, *p* < .001, whilst the addition of illness perceptions in the final step explained an additional 1.2% of the variance in depression, *ΔR*^2^ = 0.012, ns. Baseline depression score was the only significant predictor in the final model (β = .193, *p* < .05) (see Table 4).

**Table 4.** Hierarchical regression analyses predicting time 2 depression.

Step		β at step	Δ*R*^2^ for step	Total *R*^2^	95% CI
Step 1	Age	.012			−.024–.030
	Income	−094			−.121–.016
	Education	−.203**			−1.252 to −.317
	Employment	.048	.064**	.064**	−.168–.390
Step 2	Age	−.096			−.058–.014
	Income	−.085			−.117–.022
	Education	−.207***			−1.268 to −.332
	Employment	.045			−.180–.384
	Symptom duration	.192*			.003–.080
	Time since diagnosis	−.029			−.057–.039
	History of surgery	.033			−.471–.827
	Comorbidity	.063	.019	.082**	−.255–.695
Step 3	Age	−.003			−.032–.025
	Income	−.003			−.058–.052
	Education	−.130			−.516–.256
	Employment	.108			−.116–.332
	Symptom duration	.006			−.026–.037
	Time since diagnosis	.017			−.021–.055
	History of surgery	−.091			−.607–.425
	Comorbidity	.218			−.147–.582
	Baseline depression	.644***	.344***	42.6***	.542–.746
Step 4	Age	−.005			−.035–.025
	Income	−.001			−.057–.055
	Education	−.090			−.494–.314
	Employment	0.98			−.128–.325
	Symptom duration	.002			−.030–.035
	Time since diagnosis	.018			−.020–.057
	History of surgery	−.090			−.618–.438
	Comorbidity	.199			−.173–.571
	Baseline depression	.614***			.497–.731
	Consequences	.058			−.090–.205
	Timeline	−.030			−.129–.070
	Personal control	−.037			−.121–.046
	Treatment control	−.032			−.114–.050
	Identity	−.072			−.190–.045
	Concern	.019			−.098–.135
	Coherence	−.004			−.079–.071
	Emotional response	.037	.012	.438***	−.079–.153

* *p* < .05. **p < .01. ***p < .001.

### Predictors of pain intensity

Following the process outlined in the regression analysis above, demographic factors were again entered in step 1 of the multiple regression, followed by clinical factors in step 2, baseline self-reported pain intensity in step 3, and illness perceptions in step 4. For pain intensity as the outcome variable, demographic factors explained 11.1% of the variance, *ΔR*^2^ = 0.111, *p* < .001. The additional of clinical factors in step 2 only increased the percentage of variance explained by 2.3%, *ΔR*^2^ = 0.017, ns. The addition of baseline pain intensity explained a further 25.5% of the variance in pain scores, *ΔR*^2^ = 0.255, *p* < .001. When illness perceptions were entered in the final step, they accounted for an additional 1.9% of the variance in pain intensity, *ΔR*^2^ = 0.019, ns. In the final model, employment status (β = 3.12, *p* < .05), along with the illness perceptions of timeline (β = 1.44, *p* < .01), and baseline pain scores (β = .575, *p* < .001) were the significant predictors (see Table 5).

**Table 5.** Hierarchical regression analyses predicting time 2 pain intensity.

Step		β at step	Δ*R*^2^ for step	Total *R*^2^	95% CI
Step 1	Age	−.026			−.360–.309
	Income	−.882*			−.012
	Education	−11.83***			−17.66 to −5.99
	Employment	3.415	.111***	.111***	−.058–6.89
Step 2	Age	−.234			−.681–.212
	Income	−.866			−1.74–.006
	Education	−11.95***			−17.78 to −6.11
	Employment	2.975			−.536–6.49
	Symptom duration	.365			−.111–.841
	Time since diagnosis	−.171			−.761–.419
	History of surgery	−1.18			−9.22–6.86
	Comorbidity	−4.63	.023	.135***	−10.34–1.09
Step 3	Age	−.100			−.477–.276
	Income	−.433			−.1.17–.305
	Education	−5.28*			−10.35 to −.201
	Employment	3.01*			.054–5.96
	Symptom duration	.078			−.327–.482
	Time since diagnosis	.117			−.382–.617
	History of surgery	−3.87			−10.66–2.92
	Comorbidity	−1.97			−6.81–2.88
	Baseline pain	.676***	.255***	.389***	.545–.807
Step 4	Age	−.015			−.404–.374
	Income	−.336			−1.08–.414
	Education	−4.52			−9.79–.756
	Employment	3.123*			.134–6.12
	Symptom duration	.041			−.384–.466
	Time since diagnosis	.160			−.344–.663
	History of surgery	−3.82			−10.71–3.08
	Comorbidity	−2.21			−7.15–2.74
	Baseline pain	.575***			.388–.761
	Consequences	−.228			−2.19–1.73
	Timeline	1.44*			.119–2.75
	Personal control	−.111			−1.20–.977
	Treatment control	.338			−.728–1.40
	Identity	.260			−1.46–1.98
	Concern	.601			−.919–2.12
	Coherence	−.226			−1.24–.785
	Emotional response	.603	.019	.409***	−.898–2.10

* *p* < .05. **p < .01. ***p < .001.

For pain-related disability as the outcome variable, the demographic factors at step 1 accounted for 7.1% of the variance, *ΔR*^2^ = 0.071, *p* < .001. The inclusion of the clinical factors at step 2 accounted for an additional 4.4% of the variance, *ΔR*^2^ = 0.044, *p* < .05. At step 3, baseline pain-related disability score predicted an additional 19.1% of the variance in pain-related disability at follow-up, *ΔR*^2^ = 0.191, *p* < .001. The inclusion of illness perceptions accounted for an additional 5.7% of the variance in pain-related disability, *ΔR*^2^ = 0.057, *p* < .01. In the final model, employment status (β = .371, *p* < .05) and baseline pain-related disability score (β =.354, *p* < .001) were the significant predictors (see Table 6).

**Table 6.** Hierarchical regression analyses predicting time 2 pain-related disability.

Step		β at step	Δ*R*^2^ for step	Total *R*^2^	95% CI
Step 1	Age	−.011			−.042–.021
	Income	−.060			−.142–.022
	Education	−.592*			−1.14 to −.022
	Employment	.441**	.071***	.071***	.113–.769
Step 2	Age	−.032			−.074–.009
	Income	−.062			−.143–.020
	Education	−.639*			−1.18 to −.095
	Employment	.420*			.092–.748
	Symptom duration	.065**			.020–.109
	Time since diagnosis	−.070*			−.125 to −.015
	History of surgery	−.205			−.956–.546
	Comorbidity	−.187	.044*	.115***	−.721–.347
Step 3	Age	−.013			−.050–.024
	Income	−.037			−.109–.036
	Education	−.188			.683–.307
	Employment	.340*			.048–.631
	Symptom duration	.035			−.006–.075
	Time since diagnosis	−.036			−.085–.014
	History of surgery	−.225			−.892–.441
	Comorbidity	.103			−.376–.582
	Baseline disability	.507***	.191***	.113***	.386–.627
Step 4	Age	−.002			−.039–.036
	Income	−.016			−.088–.056
	Education	.065			−.441–.571
	Employment	.371*			.083–.659
	Symptom duration	.021			−.020–.062
	Time since diagnosis	−.031			−.080–.018
	History of surgery	−.315			−.975–.345
	Comorbidity	.018			−.459–.496
	Baseline disability	.354***			.200–.507
	Consequences	.020			−.171–.210
	Timeline	.122			−.002–.246
	Personal control	−.055			−.159–.049
	Treatment control	.033			−.069–.135
	Identity	.104			−.055–.263
	Concern	.054			−.092–.200
	Coherence	−.010			−.108–.088
	Emotional response	.114	.057**	.363***	−.029–.258

* *p* < .05. **p < .01. ***p < .001.

## Discussion

The present study longitudinally examined the relationship between illness perceptions and outcomes in the context of endometriosis. We aimed to examine the illness perceptions held by individuals with endometriosis and explore the relationships they have with psychological distress and pain over the course of 1 year. Our findings revealed predominantly negative illness perceptions among participants regarding their endometriosis. They anticipated various adverse outcomes linked to the condition, identified with its numerous symptoms, and believed it would persist throughout their lives. They also held negative emotional views about their condition. Moreover, participants felt they lacked personal control over the condition, although perceptions about treatment were more mixed, possibly due to varying treatment paths and effectiveness. Despite this, participants reported a good level of understanding about their condition. Our cross-sectional correlation analysis showed that illness perceptions were associated with psychological distress and pain at baseline.

Our regression analyses demonstrated that, after controlling for demographics, clinical factors, and initial levels of the outcome variables, illness perceptions did not predict anxiety and depression at 12-month follow-up. Timeline was a significant predictor of pain intensity at follow-up, but the effect was small. This implies that negative beliefs about the duration of endometriosis may exacerbate pain intensity. Additionally, combined illness perceptions were a significant predictor of changes to self-reported disability over the course of a year, explaining an additional 5.7% of the variance in disability scores at follow-up, after controlling for baseline disability levels.

Our findings suggest that while participants hold negative perceptions about their illness and that these perceptions are associated with psychological distress and pain cross-sectionally, there is limited utility of illness perceptions in predicting psychological distress and pain at 12-month follow-up. The findings highlight the importance of addressing illness perceptions in psychological support and intervention programs for individuals with endometriosis. Participants’ negative perceptions of their condition, including anticipated adverse outcomes, chronicity, and emotional distress, underscore the need for tailored psychosocial support that validates their experiences while helping them develop adaptive coping strategies (Evans et al., 2019).

With the exception of timeline predicting pain intensity at follow-up, our regression analysis suggests little effect of illness perceptions on psychological distress and pain longitudinally, which may indicate that specific interventions aimed solely at modifying illness perceptions may not be warranted or sufficient. A possible explanation is that the participants’ perceptions accurately reflect the challenging reality of living with endometriosis, including its chronic nature, complex symptomatology, and varied treatment outcomes. As such, these perceptions might not necessarily represent maladaptive beliefs but rather realistic appraisals of their condition. Given this, interventions that focus on empowering individuals to manage their condition effectively rather than challenging perceptions that may be grounded in reality may be more effective. In addition, interventions that focus on building psychological flexibility, such as Acceptance and Commitment Therapy (ACT), may be particularly beneficial in this population. ACT emphasizes accepting difficult emotions and thoughts, aligning actions with personal values, and developing mindfulness skills to improve overall well-being and has been shown to be efficacious in other chronic health conditions (Konstantinou et al., 2023). This approach could help individuals with endometriosis navigate the psychological challenges of living with a chronic condition without requiring them to alter perceptions that may reflect their lived reality. Research examining the usefulness of ACT within the context of endometriosis is therefore warranted.

There are some methodological limitations that should be noted. First, the cause dimension of illness perceptions was omitted from analysis meaning that a full exploration of how illness perceptions dimensions impact distress and pain cannot be provided. However, there is currently no consensus on the aetiology of endometriosis, so it is likely that individuals with the condition simply do not know the cause, reflected in the lack of responses to this question within the current study. Of those who did respond, there was a lack of heterogeneity, with the vast majority pinpointing genetic or biological explanations for their condition. Therefore, the “cause” illness perception may have less practical relevance in the context of endometriosis than with alternate conditions. In addition, while the Brief IPQ is easy and convenient for participants to complete, it means that each illness perception is assessed by only one item. Utilizing the full length IPQ-R (Moss-Morris et al., 2002) may have been beneficial, especially as illness perceptions have not been well studied in this population.

A further limitation is that participants were mainly recruited from social media and support groups. Individuals recruited in this way may: (i) have worsened symptomology than those not attending these groups, leading them to source support; or (ii) have more well-established support networks and coping strategies. Therefore, the generalisability of responses from this group may be limited. Additionally, we did not enquire as to our participants’ gender identity. Research has demonstrated feelings of disconnection associated with experiencing endometriosis as a non-binary or transgender individual (Eder and Roomaney, 2024), which was not explored in the current study. Furthermore, due to the scope of the current study, several potentially important variables (e.g., coping) were not included. Finally, there was a lack of diversity in participant demographics, particularly with regards to ethnicity. Future research with larger sample sizes is also needed to replicate our findings.

This study provides important insights into the illness perceptions held by individuals with endometriosis and their relationships with psychological distress and pain over time. While participants reported predominantly negative illness perceptions, these views appear to reflect the challenging realities of living with endometriosis. Although illness perceptions were associated with distress and pain cross-sectionally, and timeline predicted pain intensity longitudinally, our analysis overall showed illness perceptions had limited predictive utility at follow-up suggesting that interventions targeting illness perceptions alone may not be sufficient. Instead, empowering individuals to develop effective coping strategies and fostering psychological flexibility should be prioritized. Approaches such as ACT, which focus on acceptance, mindfulness, and values-driven action, may be particularly well-suited for addressing the complex emotional and physical challenges of endometriosis. Future research should explore the efficacy of ACT in this population.
